# A proof-of-concept study of personalized dosimetry for targeted radioligand therapy using pre-treatment diagnostic dynamic PET/CT and Monte Carlo simulation

**DOI:** 10.3389/fonc.2025.1600821

**Published:** 2025-08-04

**Authors:** Thanh Tai Duong, Danny De Sarno, Hatim Fakir, Glenn Bauman, Martin Martinov, Rowan M. Thomson, Ting-Yim Lee

**Affiliations:** ^1^ Robarts Research Institute, University of Western Ontario, London, ON, Canada; ^2^ Oncology Department, University of Western Ontario, London, ON, Canada; ^3^ Physics Department, Carleton University, Ottawa, ON, Canada; ^4^ Imaging Program, Lawson Research Institute, London, ON, Canada

**Keywords:** targeted radioligand therapy (TRT), personalized dosimetry, Monte Carlo simulation, tracer kinetics, 177Lu-PSMA-617, biological effective dose (BED)

## Abstract

**Purpose:**

Theranostics integrates diagnostic imaging (e.g., ^18^F-PSMA-1007 PET) with targeted radioligand therapy (TRT; e.g., ^177^Lu-PSMA-617), but personalized dosimetry remains challenging due to complex dose calculations. Current methods like Monte Carlo simulations are accurate but require impractical post-treatment multi-day SPECT/CT imaging. Here we establish a proof-of-concept framework using pre-treatment PET/CT to predict TRT doses via graphical analysis and Monte Carlo modeling, eliminating the need for serial imaging. Our voxel-based approach demonstrates significant dose variations in prostate cancer patients under standard TRT with a one-size-fits-all radioligand dose, enabling pre-treatment dose personalization—a critical step toward precision radiotheranostics.

**Methods:**

Dynamic PET/CT scans obtained with ^18^F-DCFPyL over 22 min from six prostate cancer patients were used in this study. Tissue time-integrated activity (TIA), that is, the total number of decays from the accumulated radioligand, was calculated as the product of the area under the curve (AUC) of an extrapolated arterial time activity curve (TAC) and the Logan distribution volume (LDV) determined by graphical analysis of tissue TAC. The resulting ^177^Lu-PSMA-617 TIA map, along with the CT-derived tissue geometry, density, and composition maps, were used to calculate the absorbed dose in the prostate tumor, overall prostate, and bone marrow in the femurs by *egs_mird*, a Monte Carlo-based absorbed dose calculation. Biological effective dose (BED) was calculated using the voxel-based absorbed dose and an extended radiobiological linear quadratic model accounting for dose rate, DNA repair, and clonal repopulation.

**Results:**

Voxel-wise LDV graphical analysis demonstrated strong linearity, with an interpatient mean *R* ^2^ of 0.999973 ± 0.000047 (mean ± SD). Using a one-size-fits-all radioligand dosing approach, significant variations in absorbed dose were observed: 10.4 ± 4.9 Gy/GBq in tumors, 5.1 ± 0.7 Gy/GBq in normal prostate tissue, and 1.0 ± 0.3 Gy/GBq in bone marrow. These variations were influenced by differences in both LDV and arterial TACs among the patients—the former due to radioligand binding avidity and the latter to tumor burden and clearance rates.

**Conclusion:**

We developed a framework for personalized TRT dose calculations using pre-treatment diagnostic PET/CT scans, eliminating the need for post-treatment SPECT/CT scans via the LDV-based method. This approach addresses variability in tumor and organ-at-risk doses from one-size-fits-all radioligand dosing, enabling optimized pre-treatment planning and integration with external beam radiation therapy (EBRT) or brachytherapy, if indicated, for precise and effective therapy. This method shows promise but requires further validation through larger studies and direct comparison with post-treatment dosimetry to confirm its accuracy.

## 1 Introduction

Theranostics is an emerging treatment modality in medicine that integrates diagnostic imaging and therapeutic delivery targeting the same biological pathway. A key example is radiotheranostics, which employs radiolabeled imaging agents (e.g., ^18^F-PSMA-1007 and ^68^Ga-PSMA-11) to identify specific targets, followed by targeted radioligand therapy (TRT) using therapeutic radiolabeled agents (e.g., ^177^Lu-PSMA-617) to deliver precise radiation doses to those targets (1–3). This approach often focuses on targeting cell surface receptors, allowing for the selective delivery of high radiation doses to tumor cells while minimizing damage to surrounding healthy tissues (4). TRT has gained FDA approval for treating neuroendocrine tumors with ^177^Lu-DOTATATE and metastatic castration-resistant prostate cancer (mCRPC) with ^177^Lu-PSMA-617 (5). The approval of ^177^Lu-PSMA-617 (Pluvicto, Novartis) was based on a phase III randomized controlled trial, which demonstrated that combining TRT with standard of care (SOC) significantly improved the survival rates in mCRPC patients compared to SOC alone (6). Other phase II randomized trials suggest benefits in the earlier stages of mCRPC (7, 8).

Personalized dosimetry is a prerequisite for safe and effective treatment with other radiation-based therapies (9) but is lacking for TRT due to challenges such as validating non-uniform dose distributions in heterogeneous media or uncertainties associated with internal dose calculations. The VISION trial (10), which resulted in the FDA approval of Pluvicto, did not incorporate dosimetry into its design, underscoring the ongoing challenges of establishing dosimetry as a routine practice in targeted radioligand therapy (TRT). Three-dimensional voxel-based dosimetry is particularly helpful in assessing radiation absorbed doses in target tissues and organs at risk (OAR) at millimeter resolution. Several dosimetric calculation methods can be used for this purpose, including the convolution of dose point-kernels (DPKs) or voxel S-factors (VSFs) with radioligand activity distribution (11, 12) and Monte Carlo (MC) simulation. Among these methods, MC simulation is considered to be the most accurate and promising approach for personalized dosimetry because of its ability to account for individualized geometry and tissue densities from CT images (13–15).

MC dose calculations for TRTs require an accurate assessment of time-integrated activity (TIA), that is, the total number of decays from the accumulated radioligand, in the tissue (16). Currently, tissue TIA is commonly calculated by fitting time–activity curves (TACs) measured by SPECT/CT imaging at multiple time points, such as 1, 24, 48, and 72 h after administering the therapeutic radioligand, and extrapolating to infinity (more correctly, the total tissue residence time) to determine the area under the curve AUC (17). In the literature, there are several studies proposing dosimetric protocols that require only a few imaging sessions after each treatment cycle (18, 19). These studies have shown that reducing the number of imaging time points is feasible but may introduce uncertainty or bias in absorbed-dose estimates. Additionally, several studies have suggested single time point (STP) imaging with assumed pharmacokinetic parameters to estimate the tissue TIA and absorbed dose (20–24). Although STP imaging offers simplicity and reduced imaging time, it may result in less accurate absorbed-dose estimates than using multiple time points (MTP). Conversely, MTP imaging can improve the accuracy but adds scheduling complexity and imaging time. Fitting four or six parameters (for a sum of double or triple decaying exponential functions) to four or less time point data is an underdetermined estimation problem as well and can lead to large variabilities in the estimates. In summary, acquiring MTP images to construct TACs can be challenging due to various constraints, such as patient compliance, technical limitations, or logistical issues. In addition, both STP and MTP imaging do not address the need for pre-treatment dosimetric planning. We, therefore, sought to predict the absorbed doses delivered by ^177^Lu-PSMA-617 using a pre-treatment ^18^F-DCFPyL PET/CT scan, which is required for treatment qualification, eliminating the need for multiple SPECT/CT scans over time post-treatment. Our method uses graphical analysis (25–28) to determine the tissue TIA, from which the voxel-based absorbed dose was estimated by an in-house MC program (13) before conversion to biological effective dose (BED) with radiobiological modeling. In this report, we first described the algorithmic pipeline and then the results from application of the pipeline to six prostate cancer patients from our retrospective database. The contribution of this work to the TRT field is twofold (1): developing a pipeline for personalized voxel-wise dosimetry using a MC simulation method based on the patient’s pre-treatment diagnostic PET/CT study and (2) investigating the extent of radiation dose variations if prostate cancer patients were treated with the standard radioligand activity, 7.4 GBq, as prescribed in the VISION trial. This study is intended as proof-of-concept exploration to assess the feasibility of personalized TRT dose estimation using diagnostic imaging data despite the fact that the pharmacokinetic similarity between the diagnostic and therapeutic radioligands in all tissues has not yet been clinically validated.

## 2 Materials and methods

**Figure 1:**
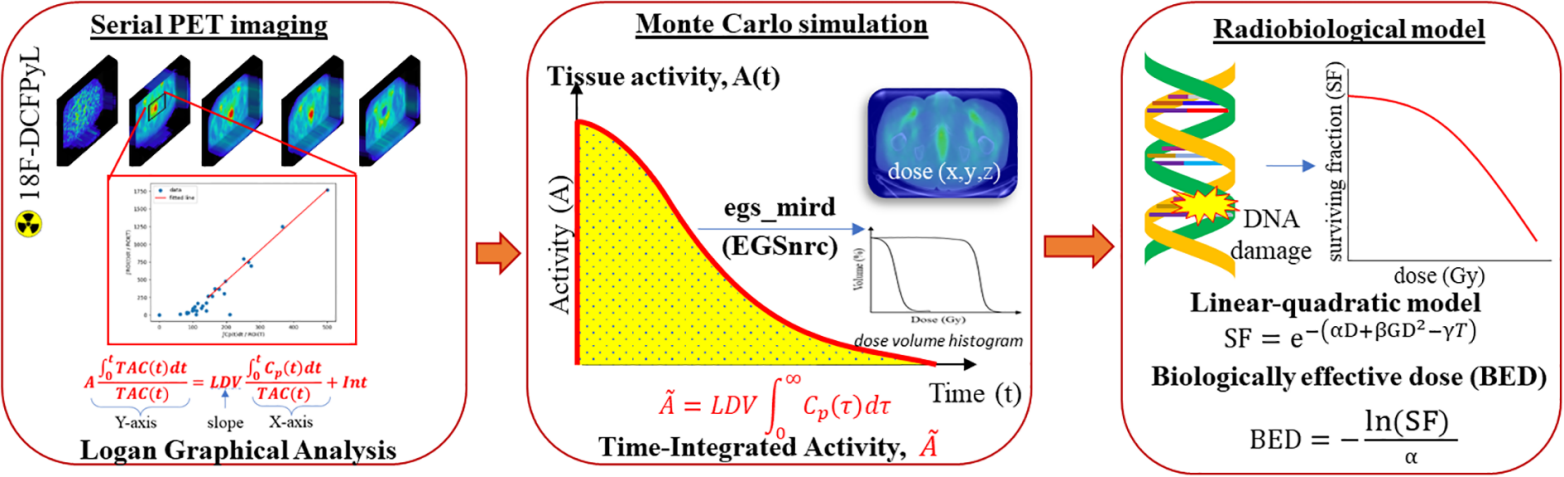
Proposed workflow for patient-specific TRT dosimetry. The symbols and equations are explained in the text.

In this study, we utilize pharmacokinetic theory and graphical analysis to obtain TIA from a single dynamic multi-phase PET/CT scan, which, to our knowledge, has not yet been studied before. We used Logan plot to determine the Logan distribution volume (LDV) per voxel (25, 26) and then multiplied the LDV with the patient’s extrapolated arterial AUC to obtain the voxelized tissue TIA. After the ^177^Lu TIA map has been determined, a full MC simulation, egs_mird (13), was carried out using the TIA data and CT images to determine the absorbed dose distributions in the prostate tumor, overall prostate, and bone marrow in the femurs. The absorbed dose is further converted into BED using a radiobiological linear quadratic model, which accounts for dose rate, DNA repair, and clonal repopulation effects. The physical radiation dose and BED were then normalized to a standard administered activity of 7.4 GBq per cycle. **Figure 1** summarizes the proposed algorithmic pipeline for patient-specific TRT dosimetry which includes the following steps (1): estimation of the tissue TIA using Logan graphical analysis from a dynamic diagnostic PET/CT study (2), calculation of the absorbed dose from the estimated TIA using a Monte Carlo program (egs_mird (13)), and (3) estimation of BED from the absorbed dose.

### 2.1 Algorithmic pipeline for patient-specific TRT dosimetry: Logan graphical analysis-derived TIA

The tissue time-integrated activity (TIA) at voxel (*x*,*y*,*z*) is calculated as Equation 1:


(1) $$\text{TIA}\left(x,y,\;z\right)=\;\int_{0}^{\infty }\text{TAC}\left(x,y,\;z,\;t\right)dt$$

where $\text{TAC}\left(x,y,z,t\right)$ is the measured activity in voxel $\left(x,y,\;z\right)$ at time *t*. The integral is from the time of injection of the therapeutic radioligand $\left(t=0\right)$ to the total activity resident time in voxel $\;\left(x,y,\;z\right)$ . This time is taken to be five times of the effective half-life of activity, $T_{1/2,eff}$ , defined as Equation 2:


(2) $$\frac{1}{T_{1/2,eff}}=\frac{1}{T_{1/2,p}}+\frac{1}{T_{1/2,b}}$$

where $T_{1/2,p}$ is the physical half-life of the radioactivity and $T_{1/2,b}$ is the biological half-life of the therapeutic radio which is dependent on its *in vivo* pharmacokinetics. Since most therapeutic radioisotopes have a $T_{1/2,p}$ close to a week (e.g., 6.7 days for ^177^Lu), it is impractical to measure $\text{TAC}\left(x,y,z,t\right)$ out to $5T_{1/2,eff}$ in clinical practice. Therefore, a common method of calculating the total *TIA* involves curve fitting the *TAC_S_* and extrapolating the fit to infinity to determine the AUC. While this method is widely used, it has limitations as discussed in the “Introduction”. Here we use pharmacokinetic theory (29, 30) and Logan graphical analysis (25, 26) of compartmental systems to determine the $TIA\left(x,y,z\right)$ . First, from pharmacokinetic theory (29, 30):


(3) $$TAC\left(x,y,z,t\right)=C_{a}\left(t\right)\;⊗R\left(x,y,z,t\right)$$

where (*t*) is the arterial input function (*AIF*) in Bq mL^-1^, $R\left(x,y,z,t\right)$ is the flow-scaled impulse residue function in min^-1^, and ⊗ is the convolution operator. In the following simplified notation, the spatial variables will be dropped with the implicit understanding that all equations apply to a discrete tissue region at $\left(x,y,z\right)$ . To obtain the TIA, we integrate Equation 3 and apply Fubini’s theorem:


(4) $$\text{TIA}=\int_{0}^{\infty }C_{a}\left(t\right)dt\cdot \int_{0}^{\infty }R\left(t\right)dt$$

Equation 4 states that TIA is equal to the product of the area under the curve (AUC) of AIF and $R\left(t\right)$ , that is,


(5) $$TIA=\left({\text{AUC}}_{C_{a}\left(t\right)}\right)\cdot \left({\text{AUC}}_{R\left(t\right)}\right)\;$$

To calculate the ${\text{AUC}}_{C_{a}\left(t\right)}$ , we fitted image-derived arterial curves and then used population data for extrapolation for the past 22 min. The details of the fitting and calculation of the ${\text{AUC}}_{C_{a}\left(t\right)}$ is discussed in Appendix 1.

**Figure 2:**
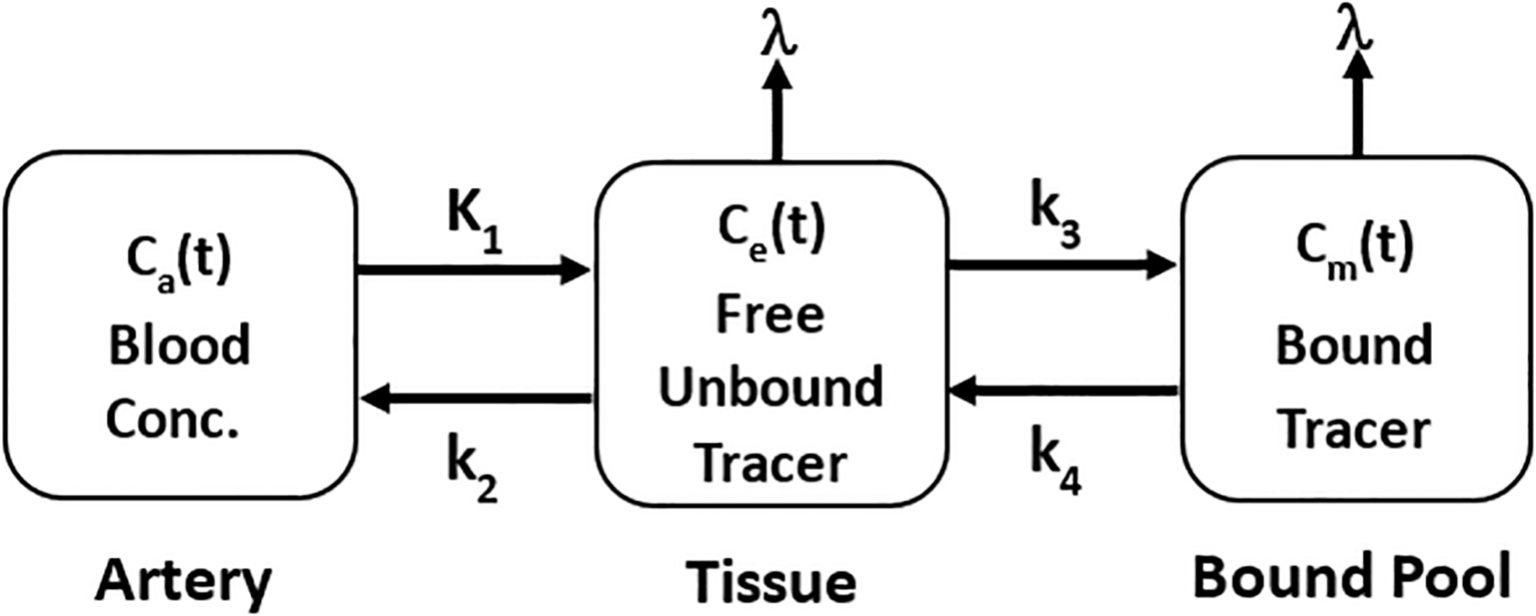
Standard two-tissue compartment model incorporating the rate of decay $\lambda \;\left[{\text{min}}^{-1}\right]$ of radionuclide for pharamacokinetics of TRT radioligands. $K_{1}\;\left[\text{mL}\cdot {\text{min}}^{-1}\cdot {\text{g}}^{-1}\right]\;$ is the transfer rate constant of radioligand from blood to tissue, $k_{2}\;\left[{\text{min}}^{-1}\right]$ is the efflux rate constant of free unbound tissue radioligand to blood, $k_{3}\;[{\text{min}}^{-1}]$ is the binding rate constant of radioligand to target, and $k_{4}\;\left[{\text{min}}^{-1}\right]$ is the dissociation rate constant of bound radioligand from target.

For TRT, the pharmacokinetics of the radiolabeled ligand can be described by modifying the standard two-tissue compartment model (S2TCM) to account for the radioactive decay of the radionuclide as shown in **Figure 2**.

In Appendix 2, it is shown that *R*(*t*) for the decay-incorporated S2TCM can be expressed as Equation 6:


(6) $$R\left(t\right)=Ge^{-\alpha t}+He^{-\beta t}$$

where G, H, α , and *β* are as defined in the Appendix and the *AUC* of $R_{f}\left(t\right)$ is given by:


(6a) $$\text{AUC}\;R\left(t\right)=\frac{G}{\alpha }+\frac{H}{\beta }=\frac{K_{1}\left(k_{4}+k_{3}+\lambda \right)}{k_{2}k_{4}+\left(k_{2}+k_{3}+k_{4}\right)\lambda +\lambda^{2}}\;$$

For therapeutic radionuclides with half-lives >6.0 days, like ^161^Tb, ^177^Lu, and ^225^Ac, $\text{λ}\ll {\text{k}}_{2},{\text{k}}_{3},{\text{k}}_{4}$ , Equation 6a is simplified to:


(7) $$\text{AUC}\;R\left(t\right)=\frac{K_{1}}{k_{2}}(1+\frac{k_{3}}{k_{4}})=\text{LDV}$$

The significance of Equation 7 in the estimation of TIA via Equation 5 is twofold: first, it is the Logan distribution volume (LDV) (25) for the S2TCM without the radioactive decay; second, it suggests that the therapeutic (e.g., ^177^Lu-PSMA-617) and diagnostic (e.g., ^18^F-DCFPyL) radioligand have the same pharmacokinetics in tissue, i.e., the same $K_{1},\;k_{i},\;i=2,3,4$ ; then, it is possible to determine the therapeutic radioligand’s AUC *R*(*t*) from a pre-treatment diagnostic study with the diagnostic radioligand using Equation 7. Finally, tissue TIA at therapy can be predicted with Equation 5; here the $\text{AUC}\;C_{a}\left(t\right)$ from the diagnostic study has to be scaled by the ratio of the administered activities for the therapeutic treatment and diagnostic study.

LDV can be determined by Logan graphical analysis (26) of the diagnostic study as follows:


(8) $$\frac{\int_{0}^{t}\text{TAC}(\tau )d\tau }{\text{TAC}(t)}=\text{LDV}\cdot \frac{\int_{0}^{t}C_{a}(\tau )d\tau }{\text{TAC}(t)}+Int$$

Equation 8 shows that LDV is the slope of the tissue-normalized cumulative area of the tissue TAC versus the tissue normalized cumulative area of a selected arterial TAC. Equation 8 is valid only after a delay time t_0_^*^ when the distribution of radioligand is in quasi-equilibrium. Our algorithm searches for the best-fit line within a delay range of ~2.33–8 min and selecting one with the maximum coefficient of determination (*R* ^2^). Note that by transforming the tissue and arterial TACs as outlined in Equation 8, the fitting for LDV and *K_i_* becomes linear. This implies that, provided quasi-equilibrium is achieved, a minimum of only two data points are required.

### 2.2 Patients’ data

Six patients were selected consecutively from our database based on three criteria: (1) complete dynamic PET/CT datasets, (2) image quality sufficient for voxel-wise graphical analysis, and (3) availability of anatomical CT data for Monte Carlo (MC) simulations. Image data was downloaded from the IGPC-2 database which contains image studies and region of interest (ROI) contours for patients enrolled in a prospective clinical trial (ClinicalTrials.gov Identifier: NCT04009174) on men with untreated, biopsy-proven localized prostate cancer. The study was approved by and conformed to the ethical standards of the institutional research ethics committee (University of Western Ontario Research Ethics Board). Following an intravenous injection of 325 MBq of ^18^F-DCFPyL, the patients underwent a 22-min dynamic PET scan covering a 16-cm pelvic region that included the prostate with a Discovery VCT PET/CT scanner (GE Healthcare, Waukesha, WI, USA). The scan was acquired in list mode at 47 contiguous axial slices (each with 3.27 mm thickness). Image reconstruction used ordered-subset expectation maximization (OSEM) with eight subsets and four iterations, producing 128 × 128 matrix multiphase images with the following temporal sequence: 11 images at 10-s frames, followed by five at 20 s, four at 40 s, four at 60 s, and four at 180 s. A CT scan of the region was also acquired for activity localization and attenuation correction. The CT scan was acquired in helical mode using the following parameters: 140 kV, 100 mA, 0.5-s gantry rotation time, and a pitch factor of 0.984. Images were reconstructed using GE Healthcare’s PET attenuation correction (AC) kernel into 47 axial slices, each 3.75-mm thick, with a 512 × 512 matrix size. The PET and CT scans of the patients were analyzed following the dosimetry pipeline illustrated in **Figure 1** to calculate voxel-based absorbed dose and BED in the prostate and OAR calculations. AIFs were derived by sampling and averaging TACs from either the left or right common iliac artery across 10 contiguous axial slices. No partial volume correction was applied to these image-derived AIFs, as any partial volume effects would have minimal impact on LDV estimation. This is because the common iliac artery’s mean diameter (~10 mm) substantially exceeds the PET/CT scanner’s spatial resolution (~5 mm), effectively minimizing resolution-related quantification errors. ROIs were drawn using MIM software (MIM Software Inc., Cleveland, OH, USA). Normal organ ROIs (e.g., bone marrow, prostate) were automatically segmented using built-in anatomical contouring tools in MIM Software based on CT imaging. For tumor ROIs, segmentation was performed manually on PSMA PET images based on 30% SUV_max_ threshold. These segmentations were reviewed and adjusted as needed by board-certified radiation oncologists.

### 2.3 Monte Carlo simulation

Dose calculations by our MC simulation-based software, egs_mird, has been previously described in detail (13). We used the TIA generated by the methods described in Sections 2.1 and 2.2, the CT scans, and the drawn ROI contours as input for our MC dose calculations. Since the 3D dose distribution calculated by egs_mird is normalized by the total sum TIA across all voxels, to obtain the 3D absorbed dose, the 3D dose from MC simulation is multiplied by the total sum TIA to obtain Gy from Gy/Bq·s. The detailed MC simulation parameters, as recommended by AAPM Task Group 268 (TG-268) (31), are provided in the **Supplementary Material** (**Appendix 4**, **Table A4.1**).

### 2.4 Biological effective dose

In this work, we used the modified linear–quadratic (LQ) model (32) to estimate cell survival fraction (SF) as a function of dose delivered (*D*), incorporating the effects of dose rate, DNA repair, and cellular repopulation (33–35) as shown in Equation 9.


(9) $$\ln\left(SF\right)=\;-\alpha D-\beta GD^{2}+\gamma \left(T_{t}-T_{k}\right)$$

where *D* is the TRT physical dose calculated by egs_mird, *α*is linear sensitivity coefficient, *β*is quadratic sensitivity coefficient, *γ* is repopulation rate, *T_t_* is the treatment time, *T_k_* is the kick-off (lag) time for repopulation, and *G* is the unitless Lea–Catcheside factor (36), described by Equation 10:


(10) $$G=\frac{2}{\mu -\lambda }{\left(\frac{\lambda }{1-e^{-\lambda T_{t}}}\right)}^{2}\left(\frac{e^{-\left(\mu +\lambda \right)T_{t}}-1}{\mu +\lambda }-\frac{e^{-2\lambda T_{t}}-1}{2\lambda }\right)$$

where *μ* is the exponential repair rate constant and *λ* is the decay rate constant of ^177^Lu.

The biologically effective dose (BED), which takes into account dose per fraction or dose rate and total dose, DNA repair, and clonal repopulation, may be a more useful metric than physical absorbed dose for assessing the relationship between tumor response and radiation. BED is defined by Equation 11:


(11) $$\text{BED}=-\frac{\left.ln(SF\right)}{\alpha }$$

Therefore, the BED for TRT is expressed as:


(12) BED(D)=D(1+D×Gαβ)−ln(2)×(Tt−Tk)αTp

where *T_p_* is the cell repopulation time and $\gamma =ln\left(2\right)/T_{p}$ .

## 3 Results

TIA was calculated as the product of LDV and AUC of the arterial curve, as described in Eq. (5). Both factors exhibit large variations among the patients. As shown in **Table 1**, the interpatient LDV (mean ± SD) in tumor, normal prostate, and right and left femurs were 2.97 ± 1.22, 1.46 ± 0.22, 0.31 ± 0.03, and 0.31 ± 0.04 mL/g, respectively, while the mean ± SD AUC of the arterial curves was 1.43 ± 0.36 × 10^8^ Bq^8^s/mL. Note that the TIA in tumor for patient IGPC-02–026 would be approximately two times larger due to its elevated mean tumor LDV of 5.38 mL/g, which correlates to approximately two times higher tumor dose as shown in **Table 2**. Voxel-wise graphical analysis for determining LDV maps involves fitting lines across multiple delays, with the delay and LDV corresponding to the highest *R* ^2^ value selected for further use. The interpatient mean ± SD of the mean maximum *R* ^2^ per patient was 0.999973 ± 0.000047, which demonstrated strong linearity. Similarly, the interpatient mean ± SD of the average mean *R* ^2^ of the LDV fits across all delays, and all patients were 0.999966 ± 0.000006 which showed the stability of the linear fit despite delay choice.

**Table 1:** LDV (mean ± SD mL/g) in regions of interest and AUC *C_a_* (t) (Bq^8^s/mL).

Patients	Tumor	Total prostate	Normal prostate	Femur R	Femur L	AUC *C_a_* (t)
IGPC-02-026	5.38 ± 2.43	1.73 ± 1.23	1.56 ± 0.79	0.26 ± 0.07	0.26 ± 0.06	1.50 × 10^8^
IGPC-02-028	2.78 ± 0.57	1.69 ± 0.94	1.67 ± 0.93	0.36 ± 0.10	0.36 ± 0.08	1.13 × 10^8^
IGPC-02-029	2.16 ± 0.22	1.19 ± 0.33	1.17 ± 0.31	0.32 ± 0.08	0.32 ± 0.06	2.00 × 10^8^
IGPC-01-031	2.72 ± 0.91	1.27 ± 0.54	1.19 ± 0.38	0.29 ± 0.09	0.27 ± 0.06	1.58 × 10^8^
IGPC-02-032	2.03 ± 0.19	1.56 ± 0.36	1.54 ± 0.35	0.30 ± 0.08	0.31 ± 0.10	1.38 × 10^8^
IGPC-02-033	2.77 ± 0.22	1.66 ± 0.47	1.63 ± 0.42	0.32 ± 0.07	0.32 ± 0.07	1.00 × 10^8^
Mean ± SD	2.97 ± 1.22	1.52 ± 0.23	1.46 ± 0.22	0.31 ± 0.03	0.31 ± 0.04	(1.43 ± 0.36) × 10^8^
Median	2.74	1.61	1.55	0.31	0.31	1.44 × 10^8^
Range	2.03–5.38	1.19–1.73	1.17–1.67	0.26–0.36	0.26–0.36	(1.00–2.00) × 10^8^

**Table 2:** Absorbed dose (Gy) in tumor and organ at risk for one cycle of 7.4 GBq.

Patients	Tumor	Total prostate	Normal prostate	Femur R	Femur L
IGPC-02-026	146.3	48.0	43.3	6.7	6.7
IGPC-02-028	57.2	35.3	35.0	7.0	6.9
IGPC-02-029	77.7	44.1	43.5	11.0	10.9
IGPC-02-031	77.4	36.8	34.6	7.7	7.2
IGPC-02-032	50.9	39.4	38.9	7.1	7.4
IGPC-02-033	50.6	30.9	30.2	5.3	5.4
Mean ± SD	76.7 ± 36.2	39.1 ± 6.2	37.6 ± 5.3	7.5 ± 1.9	7.4 ± 1.8
Median	67.3	38.1	36.9	7.1	7.0
Range	50.6–146.3	30.9–48.0	30.2–43.5	5.3–11.0	5.4–10.9

**Figure 3:**
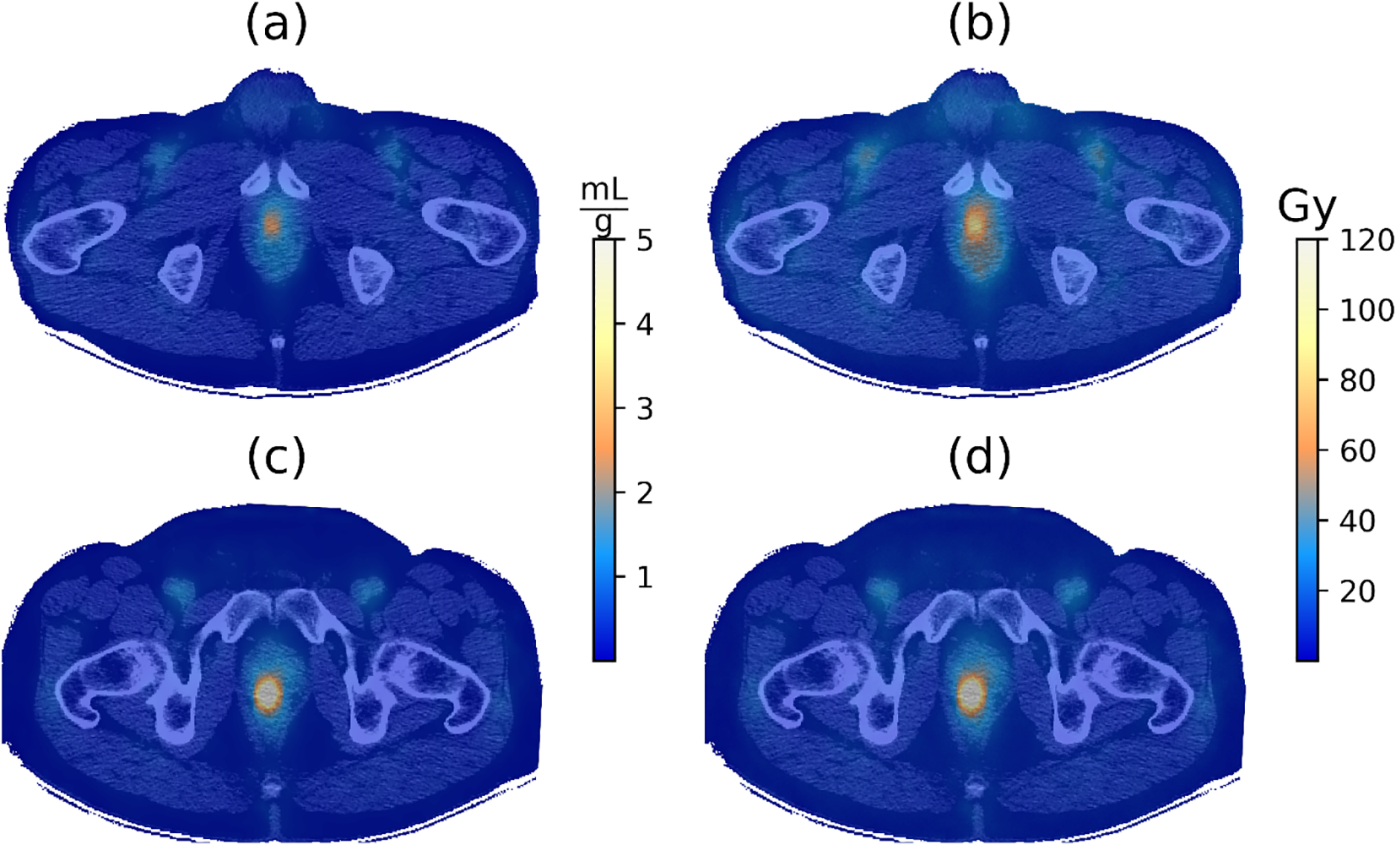
**(a)** Logan distribution volume (LDV) and **(b)** absorbed dose maps overlaid on the corresponding CT image for patient IGPC-02-029. **(c)** LDV and **(d)** absorbed dose maps overlaid on the corresponding CT image for patient IGPC-02-026. The BED was calculated using the parameters from **Table 3**.

**Figure 3** contrasts the LDV and absorbed dose maps for two patients IGPC-02–29 and IGPC-02-26. The differences in the LDV maps between the two patients, shown in **Figures 3a, c**), accounted for the majority of the disparity between the absorbed dose maps shown in **Figures 3b, d**). The remainder of the differences between the absorbed dose maps was contributed by the individual patient’s arterial input function (AIF) AUCs.

**Figure 4:**
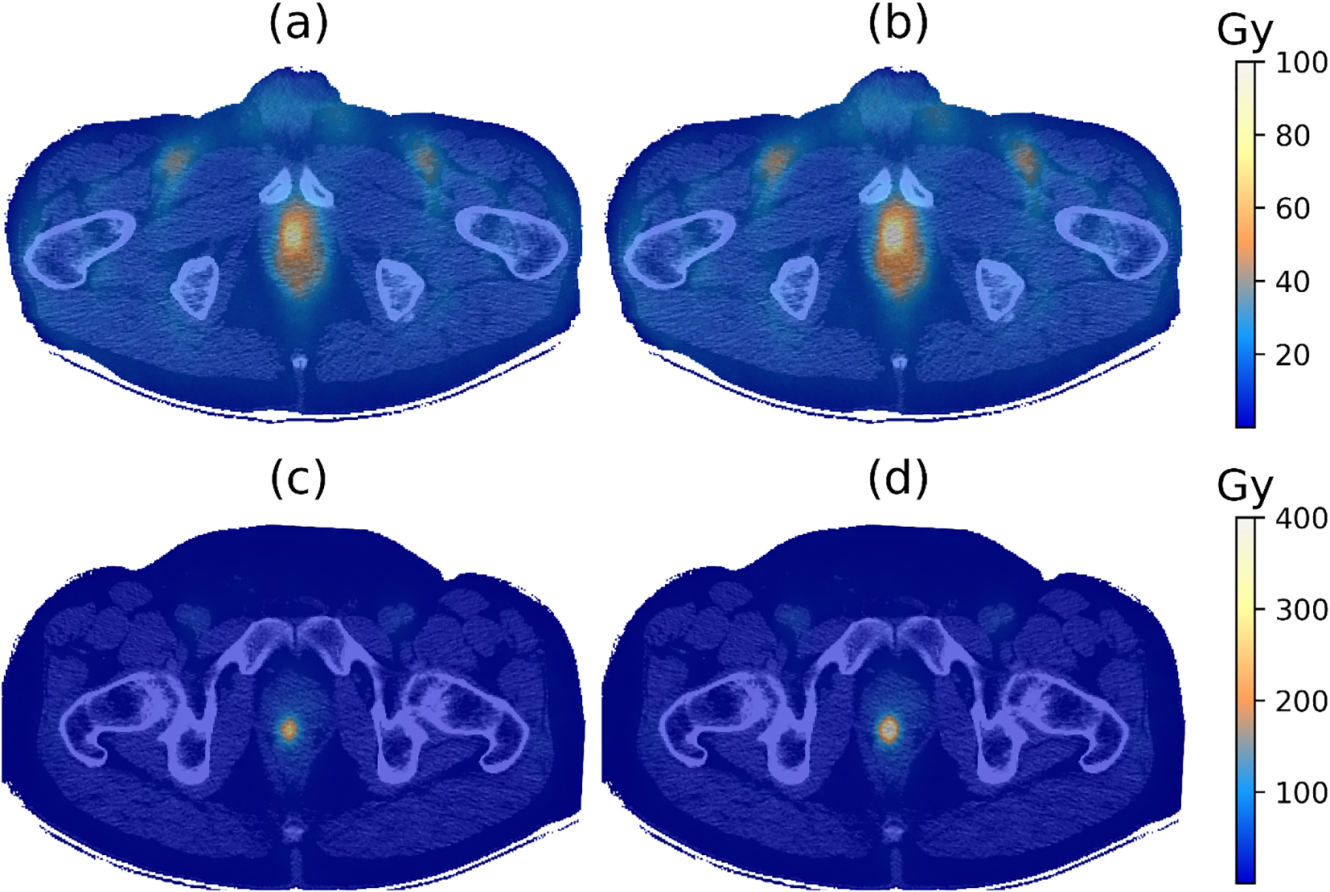
**(a)** Absorbed dose and **(b)** biological effective dose (BED) maps overlaid on the corresponding CT image for patient IGPC-02-029. **(c)** Absorbed dose and **(d)** BED maps overlaid on the corresponding CT image for patient IGPC-02-026. The BED was calculated using Equation 12 and the parameter values from **Table 3**.

**Figure 5:**
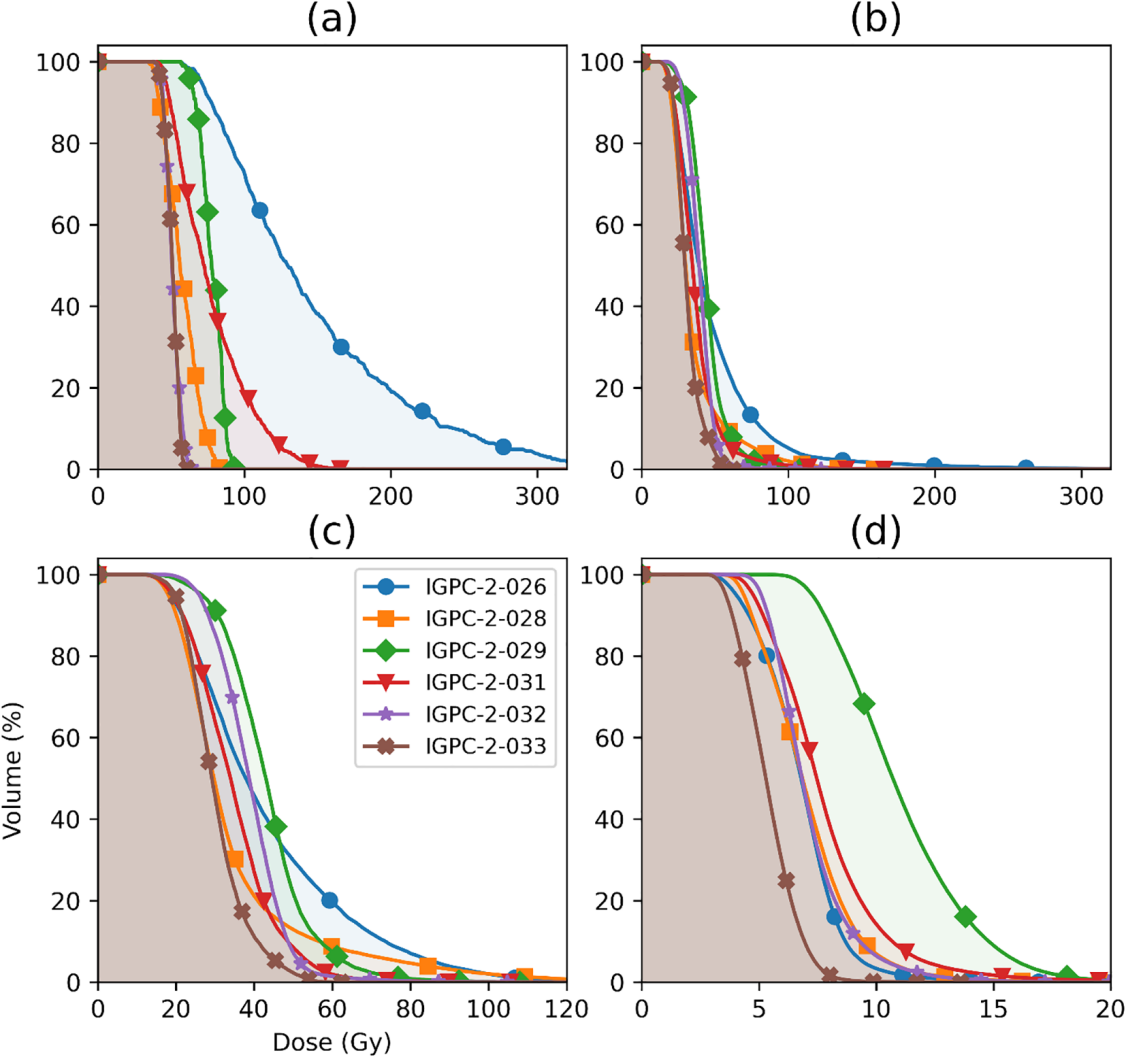
Absorbed dose volume histogram (DVH) for **(a)** tumor (30% SUVmax), **(b)** total prostate, **(c)** normal prostate (prostate minus tumor), and **(d)** bone marrow in the right femur for 7.4 GBq of injected activity.

**Figure 4** compares the absorbed dose and BED maps for the same two patients, IGPC-02–029 and IGPC-02-026. The BED maps show a higher value compared to the absorbed dose maps as expected from Equation 12 and **Table 3**. Patient IGPC-02-29 (**Figure 4a**) had a significantly more homogeneous absorbed dose distribution than patient IGPC-02-26 (**Figure 4b**). This qualitative impression was corroborated by the DVH results. **Figure 5** shows that there were significant variations in the mean and distribution of the DVH for the four different tissue types: tumor, bone marrow, whole, and normal prostate among the six patients—for example, in the tumor (**Figure 5a**), patient IGPC-02–026 has a wider distribution and higher mean than patient IGPC-02-029, but in the bone marrow (**Figure 5b**), IGPC-02–029 had the highest mean right femur bone marrow dose among the six patients. These results underline the fact that patient-specific dosimetry is required to harmonize the inter-patient BED to different tissues.

**Table 3:** Parameters used for BED calculation.

Parameters	Descriptions	Values
T_1/2_	Physical half-life	6.67 (days)
T_rep_	Repair half-life	1.5 (h) (37)
T_t_	Treatment time	33.5 (days)
T_k_	Kick-off time for tumor	56 (days) (37)
T_p_	Cell repopulation time	250 (days) (37)
α	Linear sensitivity coefficient	0.217 (Gy^-1^) (38)
αβ	Ratio α to β	3 for normal and tumor tissue (Gy) (38–40)
λ	Decay constant	0.103 (days^-1^)
μ	Repair rate	11.09 (days^-1^) (37)
$\gamma \;=\;\frac{\text{ln}\left(2\right)}{T_{p}}$	Repopulation rate	0.00277 (days^-1^) (37)

**Table 4** shows the mean, standard deviation, coefficient of variation, median, and range of the voxel-based absorbed dose in the tumor (30% SUVmax), total prostate, normal prostate (prostate minus tumor), right femur, and left femur estimated by egs_mird simulation, where the absorbed doses (Gy/GBq) were normalized from 0.33 GBq of the administered activity in the ^18^F-DCFPyL study.

**Table 4:** Absorbed dose in tumor and organ at risk (Gy/GBq).

	Tumor	Total prostate	Normal prostate	Femur R	Femur L
Mean ± SD	10.4 ± 4.9	5.3 ± 0.8	5.1 ± 0.7	1.0 ± 0.3	1.0 ± 0.2
COV (%)	47.3	15.8	14.0	25.5	24.9
Median	9.1	5.2	5.0	1.0	1.0
Range	6.9–19.8	4.2–6.5	4.1–5.9	0.7–1.5	0.7–1.5

COV, coefficient of variation.

In our study, the absorbed doses for bone marrow in femurs (Gy/GBq) were higher than the values reported in the literature, as presented in **Table 5**. It should be noted that due to the limited scan range—16 cm, the bone marrow dose in the femurs was assumed to be representative of bone marrow in the whole body for our studies. **Table 6** compares the tumor absorbed doses in Gy/GBq obtained in this study with previously published data.

**Table 5:** Comparison of the absorbed doses for bone marrow (Gy/GBq) between this study (localized prostate cancer) and previous published data (metastatic castration-resistant prostate cancer).

Study	Bone marrow (Gy/GBq)
Kabasakal et al. (2015) (41)	0.034 ± 0.01
Delker et al. (2016) (42)	0.012 ± 0.005
Kratochwil et al. (2016) (43)	0.03 ± 0.01
Scarpa et al. (2017) (44)	0.04 ± 0.03
Fendler et al. (2017) (45)	0.002 ± 0.005
Yadav et al. (2017) (46)	0.048 ± 0.05
Gosewisch et al. (2018) (47)	0.011 ± 0.002
Gosewisch et al. (2019) (48)	0.262 ± 0.24
Violet et al. (2019) (49)	0.11 ± 0.10
Kamaldeep et al. (2021) (50)	0.03 ± 0.02
Prive et al. (2021) (51)	0.02 ± 0.00
Peters et al. (2022) (52)	0.017 ± 0.008
Feuerecker et al. (2022) (53)	0.30 ± 0.27
This study	1.0 ± 0.3

**Table 6:** Comparison of the absorbed doses for tumor site (Gy/GBq) between this study and previous published data.

Study	Tumor site	Tumor dose (Gy/GBq)
Kratochwil et al. (2016) (43)	Metastases	14.05 ± 6.08
Fendler et al. (2017) (45)	Tumor	6.10 ± 4.90
Yadav et al. (2017) (46)	Tumor	10.94 ± 18.01
Scarpa et al. (2017) (44)	Tumor doses for skeletal	3.40 ± 1.94
Prive et al. (2021) (51)	Target lesion	2.14 ± 1.83
Kamaldeep et al. (2021) (50)	Primary site	3.29 ± 2.76
This study	Tumor (30% SUVmax) Whole prostate	10.4 ± 4.9 5.3 ± 0.8

**Table 2** shows the estimated mean tumor and organ doses if prostate cancer patients were treated with the standard radionuclide dose of 7.4 GBq as prescribed in the VISION trial. The mean estimated dose was 76.7 ± 36.2 Gy for tumor, 39.1 ± 6.2 Gy for total prostate, 37.6 ± 5.3 Gy for normal prostate, 7.5 ± 1.9 Gy for right femur, and 7.4 ± 1.8 Gy for left femur.

**Table 7** summarizes the BED results for the tumor and organs. Assuming a 7.4-GBq treatment, the BED was found to be 102.4 ± 65.3, 45.3 ± 8.3, 43.0 ± 6.8, 7.7 ± 2.0, and 7.6 ± 1.9 Gy for the tumor, total prostate, normal prostate (tumor minus prostate), right femur, and left femur, respectively. These results indicate that the tumor received the highest BED, while the femurs received the lowest. It is also worth noting that the ranges of physical dose and consequently BED values were quite large for all organs, indicating that there is considerable variability in delivered dose among patients.

**Table 7:** BED in ROIs for one cycle (Gy).

Patients	Tumor volumes (mL)	Tumor	Total prostate	Normal prostate	Femur R	Femur L
IGPC-02-026	1.68	230.4	59.2	51.0	6.8	6.9
IGPC-02-028	1.06	68.4	40.6	40.2	7.1	7.1
IGPC-02-029	0.71	97.7	50.9	50.1	11.4	11.3
IGPC-02-031	2.60	99.1	42.0	38.9	7.9	7.3
IGPC-02-032	1.77	59.5	44.7	44.1	7.3	7.6
IGPC-02-033	1.37	59.1	34.3	33.4	5.4	5.4
Mean ± SD	1.53 ± 0.66	102.4 ± 65.3	45.3 ± 8.3	43.0 ± 6.8	7.7 ± 2.0	7.6 ± 1.9
Median	1.52	83.0	43.3	42.1	7.2	7.2
Range	0.71–2.60	59.1–230.4	34.3–59.2	33.4–51.0	5.4–11.4	5.4–11.3

## 4 Discussions

Our results using the LDV-based TIA method revealed that the average absorbed dose in the tumor was 10.4 ± 4.9 Gy/GBq, while the absorbed doses in the total and normal prostate (prostate ROI excluding tumor ROI) were 5.3 ± 0.8 and 5.1 ± 0.7 Gy/GBq, respectively. These results suggest that TRT using ^177^Lu-PSMA-617 can deliver a high dose of radiation to the tumor but significantly a lesser dose to the immediate surrounding healthy tissues. Nevertheless, the variations of absorbed doses in tumor, normal prostate, and femurs (bone marrow) were large as measured by the coefficients of variation: 47.3%, 15.8%, and 25.5% (**Table 4**). Our finding is consistent with that of Uribe et al. (54–58), who conducted an international ^177^Lu dosimetry study and discovered that mean absorbed doses varied by up to 57.7%. The large inter-patient variations in dose means that the one-size-fits-all approach may result in tumor and OAR being underdosed or overdosed, leading to varying therapeutic efficacy and normal-tissue side effects. Personalized dosimetry using the proposed framework could improve treatment outcomes while minimizing potential adverse effects in TRT (59).

**Table 6** shows that our average tumor dose was within the range –2.14–14.05 Gy/GBq of previous studies (43–46, 50, 51). The relatively large range of tumor dose in the literature could be due to confounding factors such as the low number of patients, the lack of normalization to account for tumor size or grade affecting the avidity of radioligand, as well as the imaging and dosimetry protocols among others. Therefore, the inter-study and inter-patient variability in mean radiation delivered to the tumor makes it difficult to draw meaningful conclusions other than our methods’ potential for personalized dosimetry with pre-treatment diagnostic PET scans.

We also observed that the mean bone marrow doses in the right and left femurs (as shown in **Table 4**) were 1.0 ± 0.3 and 1.0 ± 0.2 Gy/GBq, respectively. These results are significantly higher than the previously published values (41–46, 51, 52) (ranging from 0.01 to 0.1 Gy/GBq, as presented in **Table 5**). This discrepancy can potentially be attributed to several factors including the dosimetry method employed and the difference in tumor burden associated with localized vs. metastatic lesions. Most published values have relied on blood samples and employed the traditional Committee on Medical Internal Radiation Dose (MIRD) methodology. In contrast, our study utilized MC simulation for dosimetry calculations. Gosewisch et al. (47, 48) conducted a comprehensive study comparing different dosimetry methods, including MC simulation. Their findings demonstrated that the MIRD methodology estimated an average marrow dose of 0.012 Gy/GBq, whereas MC modeling of radiation transport increased the estimate to 0.047 Gy/GBq. Recently published findings have also shown high bone marrow doses in TRT (49, 53)—for instance, Gosewisch et al. (47, 48) in 2019 reported 0.262 ± 0.240 Gy/GBq, while Feuerecker et al. (53) in 2022 reported a bone marrow dose of 0.300 ± 0.270 Gy/GBq. Assuming a typical administration of 7.4 GBq, the estimated bone marrow dose would reach approximately 7.4 Gy, significantly exceeding the commonly accepted hematologic toxicity threshold of ~2 Gy per cycle for ^177^Lu-PSMA-617 therapy. This emphasizes the importance of utilizing more accurate dosimetry methods, such as MC modeling of radiation transport, to estimate doses in TRT. This finding suggests that without individualized dosimetry, patients, especially those with limited tumor burden and slower clearance, may be at risk of marrow suppression. Furthermore, the reported values cited in these papers involved patients with metastatic castration-resistant prostate cancer, whereas our IGPC-02 patients have localized dominant intraprostatic lesions (DILs). The difference in tumor burden between localized and metastatic disease can significantly impact the bone marrow dose because of slower radioligand blood clearance in the former case, as highlighted in several previous studies (49, 60). Considering these findings, several solutions should be noted for patients without disseminated disease. First, since ^177^Lu-PSMA-617 is cleared from the blood through glomerular filtration in the kidneys, administering a diuretic agent could promote diuresis and enhance its clearance. Second, reducing the administered activity per treatment cycle could also be considered. Lastly, combining TRT with other therapies, e.g., EBRT (40), may offer a potential solution to address bone marrow doses. However, these preventive measures can only be implemented if a patient-specific dose calculation is performed before treatment. Our dose calculation framework, based on pre-treatment diagnostic scans, is well suited for this purpose. Although MC simulations are widely regarded as the gold standard for dose calculations in radiation therapy, they are not immune to dosimetric uncertainties. These uncertainties may arise from multiple sources, including limitations in source geometry modeling, voxel resolution, material composition, and statistical variance associated with particle histories. The egs_mird code has been validated using Fano tests and full patient simulations, demonstrating dose estimation uncertainties below 1% in regions of interest (ROIs), including the bone marrow (13).

By considering the fractionation and repair of DNA damage in cells, BED is a valuable tool for comparing different radiation modalities with varying dose rates, fractionation schedules, tumor control probability (TCP), and normal tissue complication probability (NTCP). The BED results are summarized in **Table 7**. For 7.4 GBq of ^177^Lu-PSMA-617 administered, the BED for the tumor, total prostate, normal prostate (tumor minus prostate), right femur, and left femur were found to be 102.4 ± 65.3, 45.3 ± 8.7, 43.0 ± 6.8, 7.7 ± 2.0, and 7.6 ± 1.6 Gy, respectively. Several studies evaluating BED for ^177^Lu-PSMA therapy have been conducted (61, 62), including one by Begum et al. that simulated the impact of PSMA-positive total tumor volume on BEDs in metastatic castration-resistant prostate cancer patients (61, 62). However, they found a range of tumor BEDs from 22 ± 15 to 11.0 ± 6.0 Gy, with a BED for red marrow of 0.17 ± 0.05 to 0.32 ± 0.11 Gy for an average injected activity of 7.3 ± 0.34 GBq. Begum et al. indicated a decrease in BED with an increase in total tumor volume (TTV) within a range of 0.1 to 3 L, while our study encompassed a much smaller TTV of 1.53 ± 0.66 mL. For a meaningful comparison, we extrapolated their results relating BED and TTV to derive a mean BED of 67.25 ± 2.36 Gy based on our TTV values. Notably, this value was within our mean BED in tumor results of 102.4 ± 65.3 Gy. We used radiobiological and determined parameters as well from Gholami et al. (37, 39) for our BED calculations in normal and tumor regions. There are not many TRT studies where BED was calculated, and as such, further investigation is necessary.

Recent findings from the LuTectomy study (63) have provided additional context for understanding dose variability in TRT. This study administered one to two cycles of 7.5 GBq ^177^Lu-PSMA-617 prior to radical prostatectomy and found a range of estimated absorbed doses across different tumor regions. Their histopathologic analysis revealed variable biologic effects in prostate specimens, suggesting that heterogeneous dose deposition influences differential biological responses. In comparison, our study also observed substantial variability in tumor BED values, which aligns with the findings from LuTectomy. This reinforces the concept that dose heterogeneity can impact biological outcomes, emphasizing the necessity of personalized dosimetry in TRT planning. While the LuTectomy study provided pre-surgical dosimetry data, our study extends the investigation by analyzing BED variations in different tissue regions, including the total prostate, normal prostate, and adjacent structures.

The feasibility of using graphical analysis-derived LDV for dosimetry calculation was supported by three key observations in this study (1): the tumor mean doses aligned with previously reported ranges (**Table 6**) (2), the LDV graphical analysis was confined to a narrow range of delayed times, and (3) the Logan plot fits exhibited strong linearity with *R* ^2^ of 0.999973 ± 0.000047 and stability with *R* ^2^ of 0.999966 ± 0.000006 across all delays.

This study, as a preliminary proof-of-concept, has limitations. It is a critical assumption that the pharmacokinetics including binding potential of the diagnostic and therapeutic radioligands are the same. In our study, the diagnostic radioligand, ^18^F-DCFPyL, and the therapeutic radioligand, ^177^Lu-PSMA-617, are different molecules. As a result, using the diagnostic study to predict the therapeutic dose may introduce inaccuracies. To address this limitation, future studies could consider replacing DCFPyL with PSMA-1007 or PSMA-11, which share a closer chemical structure to PSMA-617. This similarity could lead to more comparable pharmacokinetics, improving the reliability of dose predictions. Applying our method to calculate bladder and rectum dose can be difficult without modification as the TAC of both cannot be estimated with any accuracy. To include an estimation of radiation dose to both in our current calculation formalism, we would calculate the time-integrated activity (TIA) by determining the fraction of excreted activity (in urine/feces) and modeling the residence time in each organ. We then input these TIA values into our established dose-calculation framework. Validation of our approach using window of opportunity study designs such as the LuTectomy trial could provide further biologic validation of our approach.

## 4 Conclusion

We have developed a framework for personalized dose calculations in TRT using pre-treatment diagnostic PET/CT scans. A key advantage of this approach is the use of the LDV-based method, which eliminates the need for multiple post-treatment SPECT/CT scans to determine TIA for MC dose calculations. Access to personalized dose and BED calculations before treatment could significantly enhance pre-treatment planning. This is particularly important, as we have demonstrated that the current one-size-fits-all activity dosing leads to substantial variabilities between patients in tumor and OAR absorbed doses. Pre-treatment dose calculation would facilitate the integration of TRT with other radiation treatment modalities, for example, EBRT and brachytherapy, offering a more effective strategy to maximize tumor dose delivery while minimizing radiation exposure to healthy tissues. This method shows promise but requires further validation through larger studies and direct comparison with post-treatment dosimetry to confirm its accuracy.

## Data Availability

The original contributions presented in the study are included in the article/**Supplementary Material**. Further inquiries can be directed to the corresponding author.
