# Supplementary material for: A proof-of-concept study of personalized dosimetry for targeted radioligand therapy using pre-treatment diagnostic dynamic PET/CT and Monte Carlo simulation
Source: Front Oncol. 2025 Aug 4;15:1600821. doi: 10.3389/fonc.2025.1600821 (PMC12358388; doi:10.3389/fonc.2025.1600821)
Supplement: Supplementary file 1 [file DataSheet1.docx]

**SUPPLEMENTARY MATERIAL**

**Appendix 1**

**Determination of the AUC of the Arterial TAC**

Extrapolation of the image-derived arterial input function $C_{a}(t)$ from a 22-min dynamic scan to times greater than five half-lives of ^177^Lu requires additional processing to improve its accuracy. To this end, the patients’ fitted arterial curves in conjunction with the average curve from blood samples collected over 5-8 days in 24 patients published by Baum et al (1) were used to perform the extrapolation. Briefly, it consisted of the following steps:

1. A sum of three decaying exponentials was fitted $C_{a}(t)$ from the peak to end of image acquisition (22 min): $C_{a}\left( t \right)\approx A_{o}e^{-\psi_{o}t}+A_{1}e^{-\psi_{1}t}+A_{2}e^{-\psi_{2}t}$ where $\psi_{o}>\psi_{1}>\psi_{2}$
2. Beyond 22 min, it was assumed that $C_{ae}(t)$, the extrapolated $C_{a}(t)$, was parametrized by $\psi_{1}$, $\psi_{2}$, $A_{1}^{'}$ and $A_{2}^{'}$: $C_{ae}\left( t \right)=A_{1}^{'}e^{-\psi_{1}(t-22)}+A_{2}^{'}e^{-\psi_{2}(t-22)}$. To ensure continuity at 22 min:

$$A_{1}^{'}+A_{2}^{'}=\left[ A_{1}e^{-\psi_{1}t}+A_{2}e^{-\psi_{2}t} \right]_{t=22min} (A1.1)$$

The fraction of area contributed by the exponential $A_{1}^{'}e^{-\psi_{1}t}$ to the total area from 22 min to infinity is:

$$F_{A1}=\frac{{A_{1}^{'}}/{\psi_{1}}}{{A_{1}^{'}}/{\psi_{1}}+{A_{2}^{'}}/{\psi_{2}}}$$

1. The average blood curve from Baum et al (1) was fitted to a bi-exponential function from the peak: $B_{1}e^{-\theta_{1}t}+B_{2}e^{-\theta_{2}t}$. The fraction of area contributed by the exponential $B_{1}e^{-\theta_{1}t}$ to the total area to infinity is:

$$F_{B1}=\frac{{B_{1}}/{\theta_{1}}}{{B_{1}}/{\theta_{1}}+{B_{2}}/{\theta_{2}}}$$

1. Set $F_{A1}=F_{B1}$:

$$\frac{{A_{1}^{'}}/{\psi_{1}}}{{A_{1}^{'}}/{\psi_{1}}+{A_{2}^{'}}/{\psi_{2}}}=F_{B1} (A1.1)$$

Note that if $F_{A2}$ and $F_{B2}$ are the fractions of the area contributed by the exponentials of $A_{2}^{'}e^{-\psi_{2}t}$ and $B_{2}e^{-\theta_{2}t}$ to the total area from 22 min to infinity of $C_{ae}\left( t \right)$ and the blood curve from Baum et al’s Supplementary (1) respectively, then

$F_{A1}=F_{B1} \Rightarrow F_{A2}=F_{B2}$

1. Set $\psi_{1}=2.5 h^{-1}$ and $\psi_{2}=30 h^{-1}$ to match the median short and long decay constants from Baum et al’s results (2).
2. Determine $A_{1}^{'}$ and $A_{2}^{'}$ by solving Eqs (A1.1 & 2) together.
3. The AUC of the arterial input curve, $AUC C_{a}(t)$, was calculated as the sum of three parts:

$$AUC C_{a}\left( t \right)={AUC}_{1}+{AUC}_{2}+{AUC}_{3}$$

where:

$AUC_{1}$ is the trapezoidal area under $C_{a}(t)$ until the peak

$AUC_{2}$ is the area underneath the fitted $C_{a}(t)$ to 3 decaying exponentials from the peak to end of image acquisition (22min): $\sum_{i=1}^{3} \left( {A_{i}}/{\lambda_{i}} \right)\left( 1-e^{-22\lambda_{i}} \right)$

$AUC_{3}$ is the area underneath $C_{ae}\left( t \right)$ from 22 min to infinity: ${A_{1}^{'}}/{\psi_{1}}+{A_{2}^{'}}/{\psi_{2}}$

**Table A1.1:** shows the values of $AUC_{1,2,3}$ and the percentages each contributed to $AUC C_{a}\left( t \right)$ for each of the six patients included in this study [all in units of $Bq\cdot s/mL$]:

| Patient | ${AUC}_{1}(\%)$ | ${AUC}_{2}(\%)$ | ${AUC}_{3}(\%)$ |
| --- | --- | --- | --- |
| 26 | 6.40×10^5^ (0.4) | 9.91×10^6^ (6.6) | 1.40×10^8^ (93.0) |
| 28 | 5.64×10^5^ (0.5) | 7.60×10^6^ (6.7) | 1.05×10^8^ (92.8) |
| 29 | 5.07×10^5^ (0.3) | 1.14×10^6^ (5.7) | 1.87×10^8^ (94.0) |
| 31 | 7.62×10^5^ (0.5) | 9.43×10^6^ (6.0) | 1.48×10^8^ (93.6) |
| 32 | 8.13×10^5^ (0.6) | 8.10×10^6^ (5.9) | 1.29×10^8^ (93.5) |
| 33 | 2.43×10^5^ (0.2) | 6.04×10^6^ (6.0) | 9.42×10^7^ (93.7) |

Table A1.1 demonstrates that the largest contribution of area comes from $AUC_{3}$ for all patients. We also used a range of values for each decay rate to provide an estimation of the potential individual patient variation. Ranges used were $\psi_{1}\in\left[ 1, 4 \right]h^{-1}$, and $\psi_{2}\in\left[ 10, 90 \right]h^{-1}$ Baum et al (1).

**Appendix 2**

**Pharmacokinetics of the Standard 2 Tissue Compartment Model (S2TCM) with Radionuclide Decay**

Referring to Figure 2, the pharmacokinetics of the S2TCM with radionuclide decay can be described by the following first order ordinary differential equations which can be set up by conservation of mass in each compartment:

$$\frac{dC_{e}(t)}{dt}=K_{1}C_{a}\left( t \right)-\left( k_{2}+k_{3}+\lambda\right)C_{e}\left( t \right)+k_{4}C_{m}\left( t \right) (A2.1)$$

$$\frac{dC_{m}(t)}{dt}=k_{3}C_{e}\left( t \right)-\left( k_{4}+\lambda\right)C_{m}\left( t \right) (A2.2)$$

$$Q\left( t \right)={V_{b}C}_{a}\left( t \right)+C_{e}\left( t \right)+C_{m}\left( t \right) (A2.3)$$

Using Laplace transforms (2), the impulse residue function, $R(t)$ is determined to be:

$$R\left( t \right)=Ge^{-\alpha t}+He^{-\beta t} (A2.4)$$

where:

$$\alpha=\frac{\left( k_{2}+k_{3}+k_{4}+2\lambda\right)+\sqrt{\left( k_{2}+k_{3}+k_{4}+2\lambda\right)^{2}-4\left[ k_{2}k_{4}+\left( k_{2}+k_{3}+k_{4} \right)\lambda+\lambda^{2} \right]}}{2} (A2.5a)$$

$$\beta=\frac{\left( k_{2}+k_{3}+k_{4}+2\lambda\right)-\sqrt{\left( k_{2}+k_{3}+k_{4}+2\lambda\right)^{2}-4\left[ k_{2}k_{4}+\left( k_{2}+k_{3}+k_{4} \right)\lambda+\lambda^{2} \right]}}{2} (A2.5b)$$

$$G=\frac{K_{1}\left( \alpha_{d}-k_{3}-k_{4}-\lambda\right)}{\alpha_{d}-\beta_{d}} (A2.5c)$$

$$H=\frac{K_{1}\left( k_{3}+k_{4}+\lambda-\beta_{d} \right)}{\alpha_{d}-\beta_{d}} (A2.5d)$$

**Appendix 3**

**The reliability of LDV estimated from a 22-minute dynamic PET scan.**

Due to the lack of experimental data, we assessed the reliability of Logan Distribution Volume (LDV) estimates using computer simulations. Ten tissue time-activity curves (TACs) were generated by convolving the femoral artery TAC of Patient IGPC-02-26 with flow-scaled impulse residue functions, R(t) (Eq (3) & (6)). The S2TCM model parameters (K_1_, k_2_, k_3_ and k_4_) were randomly sampled from published values (70). As derived in Eq (7), the area-under-the-curve of R(t) approximates LDV for S2TCM without radioactive decay; thus, decay constant (λ) was excluded from simulations.

Each simulated TAC was analyzed for four scan durations: 22 minutes (study protocol), 110 minutes, 220 minutes, and 6 days. The femoral artery TAC was extrapolated to longer durations using the method in Appendix 1.

Table A3.1 compares ground truth LDV values with estimates from simulated scans:

**Table A3.1**. Ground truth LDV (mL/g) vs Estimated LDV Across Scan Durations

| **Parameter set** | **Ground truth LDV** | **Estimated LDV** | | | | **22 min % error** |
| --- | --- | --- | --- | --- | --- | --- |
|  |  | **22 min** | **110 min** | **220 min** | **6 days** |  |
| 1 | 0.86 | 0.76 | 0.86 | 0.86 | 0.86 | -12.0 |
| 2 | 1.77 | 1.68 | 1.77 | 1.77 | 1.77 | -5.4 |
| 3 | 1.10 | 0.92 | 1.10 | 1.10 | 1.10 | -16.0 |
| 4 | 0.75 | 0.72 | 0.75 | 0.75 | 0.75 | -3.9 |
| 5 | 1.50 | 1.45 | 1.50 | 1.50 | 1.50 | -3.3 |
| 6 | 1.57 | 1.55 | 1.57 | 1.57 | 1.57 | -0.7 |
| 7 | 0.53 | 0.51 | 0.53 | 0.53 | 0.53 | -4.0 |
| 8 | 1.11 | 1.07 | 1.11 | 1.11 | 1.11 | -3.6 |
| 9 | 1.25 | 1.25 | 1.25 | 1.25 | 1.25 | -0.5 |
| 10 | 2.77 | 2.70 | 2.77 | 2.77 | 2.77 | -2.7 |
| **Average (SD)** | | | | | | **-5.2 (5.0)** |

This simulation study showed that:

- LDV estimates from scans ≥110 minutes showed negligible error (≤0.7%) versus ground truth.
- The 22-minute protocol underestimated LDV by **5.2 ±5.0%** on average, leading to proportional dose underestimation.

While shorter scans (22 minutes) introduce modest LDV underestimation, they remain practical for clinical use. Longer acquisitions improve accuracy but are often not feasible due to the burden on patients.

**Appendix 4**

**Table A4.1:** Summary of the MC simulation parameters

| **Parameter** | **Description** | **Reference** |
| --- | --- | --- |
| Code | EGSnrc (CLRP fork, commit c4beb16)  egs_mird (commit d5760e2) | (3) - (5) |
| Validation | EGSnrc/ egs_mird validation | egs_mird was validated through S-value cross-comparisons with published data for radionuclides including I-131, Y-90, Lu-177 as described in Martinov et al. (2022). Additional self-validation was performed using a histogram variant of the electron Fano test to confirm internal consistency of electron transport and energy deposition |
| DICOM CT Data | 47 slices of 512 × 512 voxels with 0.98 x 0.98 mm pixel spacing and 3.27 mm slice thickness |  |
| Phantom | Type: egsphant; (Patient-specific voxelized geometry from DICOM-CT using egsphant format) |  |
| Source | Lu-177, initiated from patient-specific LDV-based activity map via egs_radionuclide_source | (6) |
| Simulation parameters | Photons were transported down to 1 keV (PCUT = 0.001 MeV). Track length scoring was enabled. All other parameters were set to EGSnrc defaults. Electron transport was not modelled |  |
| Cross-sections | Pre-calculated mass-energy absorption cross sections distributed with EGSnrc were used. |  |
| Histories (statistical uncertainty with parameter k = 1) | 1 × 10^9^ histories (1 batch; 1 chunk; statistical uncertainty estimated via batch method) | This study |
| Scored quantities | Collision kerma was scored in each voxel using the photon track length estimator; no electron transport was modeled. | (7) |
| Timing | Approximately 15 min on AMD Ryzen 9 5900X (12 cores) | This study |
| Postprocessing | BED calculated voxel-wise using LQ model | This study |

**References**

1. Baum RP, Kulkarni HR, Schuchardt C, Singh A, Wirtz M, Wiessalla S, Schottelius M, Mueller D, Klette I, Wester H-J. 177Lu-Labeled Prostate-Specific Membrane Antigen Radioligand Therapy of Metastatic Castration-Resistant Prostate Cancer: Safety and Efficacy. *Journal of Nuclear Medicine* (2016) 57:1006–1013. doi: 10.2967/jnumed.115.168443.
2. Himmelstein KJ. Compartmental models and their application. By Keith Godfrey. Academic Press Inc., 24–28 Oval Road, London NWI 7Dx, England. 1983. 293 pp. 15.5 × 23.5 Price \50.00 (\32.00). *Journal of Pharmaceutical Sciences* (1984) 73:1018–1018. doi: 10.1002/jps.2600730753
3. Martinov MP, Opara C, Thomson RM, Lee T-Y. Fast beta-emitter Monte Carlo simulations and full patient dose calculations of targeted radionuclide therapy: introducing egs_mird. Med Phys. (2022) 49:6137–49. doi: 10.1002/mp.15786.
4. Kawrakow I, Rogers D, Mainegra-Hing E, Tessier F, Townson R, Walters B. EGSnrc toolkit for Monte Carlo simulation of ionizing radiation transport. *National Research Council of Canada* doi: 10.4224/40001303
5. clrp-code/EGSnrc_CLRP: The CLRP fork of EGSnrc containing additions to egs++ and egs_brachy, an egs++ brachytherapy application. (2024) https://github.com/clrp-code/EGSnrc_CLRP [Accessed May 21, 2024]
6. EGSnrc C++ class library: EGS_RadionuclideSpectrum Class Reference. https://nrc-cnrc.github.io/EGSnrc/doc/pirs898/classEGS__RadionuclideSpectrum.html [Accessed March 22, 2025]
7. Thomson RM, Taylor REP, Chamberland MJP, Rogers DWO. Reply to Comment on “egs_brachy: a versatile and fast Monte Carlo code for brachytherapy.” *Phys Med Biol* (2018) 63:038002. doi: 10.1088/1361-6560/aa9ea2
